# Development of an initiatives package to increase children’s vegetable intake in long day care centres using the Multiphase Optimisation Strategy (MOST) randomised factorial experiment

**DOI:** 10.1017/S136898002300174X

**Published:** 2023-12

**Authors:** Lucinda K Bell, Samantha Morgillo, Dorota Zarnowiecki, Claire Gardner, Shalem Leemaqz, Jennifer Arguelles, Astrid AM Poelman, Maeva O Cochet-Broch, David N Cox, Rebecca K Golley

**Affiliations:** 1 Caring Futures Institute, College of Nursing and Health Sciences, Flinders University, Bedford Park South Australia, Adelaide, SA 5001, Australia; 2 SAHMRI Women and Kids, South Australian Health and Medical Research Institute, North Terrace, Adelaide, SA, Australia; 3 Nutrition Australia Victorian Division, Carlton, VIC, Australia; 4 Commonwealth Scientific and Industrial Research Organisation (CSIRO), Health and Biosecurity, Westmead, NSW, Australia; 5 Commonwealth Scientific and Industrial Research Organisation (CSIRO), Agriculture & Food, North Ryde, NSW, Australia; 6 Commonwealth Scientific and Industrial Research Organisation (CSIRO), Health and Biosecurity, Adelaide, SA, Australia; 7 Research and Innovation Services, University of South Australia, Mawson Lakes, SA, Australia

**Keywords:** Children, Vegetable intake, Multiphase Optimisation Strategy, Long day care, Complex interventions

## Abstract

**Objective:**

To inform a package of initiatives to increase children’s vegetable intake while in long day care (LDC) by evaluating the independent and combined effects of three initiatives targeting food provision, the mealtime environment and the curriculum.

**Design:**

Using the Multiphase Optimisation Strategy (MOST) framework, a 12-week, eight-condition (*n* 7 intervention, *n* 1 control) randomised factorial experiment was conducted. Children’s dietary intake data were measured pre- and post-initiative implementation using the weighed plate waste method (1× meal and 2× between-meal snacks). Vegetable intake (g/d) was calculated from vegetable provision and waste. The optimal combination of initiatives was determined using a linear mixed-effects model comparing between-group vegetable intake at follow-up, while considering initiative fidelity and acceptability.

**Setting:**

LDC centres in metropolitan Adelaide, South Australia.

**Participants:**

32 centres, 276 staff and 1039 children aged 2–5 years.

**Results:**

There were no statistically significant differences between any of the intervention groups and the control group for vegetable intake (all *P* > 0·05). The *curriculum with mealtime environment* group consumed 26·7 g more vegetables/child/day than control (ratio of geometric mean 3·29 (95 % CI 0·96, 11·27), *P* = 0·06). Completion rates for the curriculum (> 93 %) and mealtime environment (61 %) initiatives were high, and acceptability was good (4/5 would recommend), compared with the food provision initiative (0–50 % completed the menu assessment, 3/5 would recommend).

**Conclusion:**

A programme targeting the curriculum and mealtime environment in LDC may be useful to increase children’s vegetable intake. Determining the effectiveness of this optimised package in a randomised controlled trial is required, as per the evaluation phase of the MOST framework.

Vegetables are an important source of essential nutrients and are associated with a lower risk of chronic disease^(1)^. Dietary patterns, eating habits and food preferences, including a liking for and acceptance of vegetables, are formed during early childhood^(2–5)^. However, children fail to meet dietary guideline targets for vegetables globally^(6,7)^. For example, only 6·3 % of Australian children aged 2–17 years eat the recommended amount of vegetables as advised in the Australian Dietary Guidelines, with compliance falling from 18·5 % in 2–3 year olds to 3·8 % in 4–8 year olds^(8)^. Although a plethora of studies exist to facilitate increases in children’s vegetable intake in the early years^(4)^, it remains a public health challenge to achieve meaningful and sustained increases in children’s vegetable consumption. Evidence-based strategies that are translatable into practice and feasible in real-world settings where children spend their time are required.

Early childhood education and care (ECEC) services, comprising long day care (LDC), preschool, family day care and Outside School Hours Care, are opportunistic settings to deliver public health nutrition interventions to improve children’s vegetable intake^(9)^. Participation in ECEC services increases throughout the early years, peaking at 4 years of age when up to ∼90 % of children in Australia, the USA and United Kingdom attend some form of ECEC^(10,11)^. In Australia, LDC is the main type of ECEC, making up 69 % of total ECEC services and caring for ∼60 % of children aged birth to 5 years for an average of 30 h per week^(12,13)^. Data from two Australian states indicate that in the majority of LDC centres (∼70–80 % of LDC centres in South Australia (Unpublished, Egan & Cox 2015), and New South Wales^(14)^) food is prepared and cooked on-site by a cook or chef^(15)^, contributing to ∼40–60 % of children’s daily food intake^(16,17)^. Although there are National Quality Standards^(18)^ to ensure the food and drinks provided to Australian children are ‘nutritious and appropriate’, previous studies have found that menus do not typically comply with food group recommendations^(19)^, including the recommendations for vegetable provision^(20)^. Although interventions in ECEC settings have been able to deliver improvements in children’s dietary intake, to date, most have focused on targets such as general healthy eating policies and food provision, including menu planning^(9,21)^. Furthermore, most have evaluated the impact on combined vegetable and fruit consumption rather than vegetable intake alone^(9,21)^. Given that improvements in fruit and vegetable intakes in the school setting have been largely attributed to increases in fruit consumption and not vegetable consumption^(22)^, it is important to evaluate the effect on vegetable intakes separately.

An umbrella review of interventions in ECEC settings found that the most effective interventions for promoting healthy eating were multi-component and multi-level, targeting both environmental and individual-level influences such as centre food provision, educator mealtime practice and the childcare curriculum^(9)^. Yet multi-component, multi-level interventions can be resource intensive and costly which may be a barrier for adoption into practice. The Multiphase Optimisation Strategy (MOST) framework provides an efficient approach that embeds feasibility and effectiveness within its design to reduce such barriers to adoption into practice at the intervention design stage, ideal for progression into real-world settings and scale up^(23)^. This framework comprises three phases: 1. preparation, 2. optimisation and 3. evaluation, whereby the preparation phase lays the foundation for the latter phases^(24)^.

The present study used the MOST framework to develop a multi-component intervention, that is, a package of initiatives, for the LDC setting. The preparation phase developed three initiatives based on best practice for increasing children’s vegetable intake^(25)^. The focus of the current optimisation phase study (Phase 2) is the testing of main and synergistic effects of intervention components to identify the most effective and feasible version of an intervention (i.e. optimised intervention) to take forward to test in the evaluation phase (Phase 3) using a randomised controlled trial design^(23,26)^. Thus, the present study aimed to (1) evaluate the independent and combined effects of three initiatives – targeting (1) food provision, (2) mealtime environment (i.e. staff mealtime practices) and (3) curriculum, to identify a package of initiatives that delivers an optimal increase children’s vegetable intake while in LDC and (2) undertake a process evaluation to understand acceptability and factors that influence adoption of the initiatives. The optimisation criterion was defined as the initiative package with the greatest meaningful effect on vegetable intake, taking into consideration process evaluation findings^(27)^. It was hypothesised that an intervention utilising all three initiatives would have the largest effect on vegetable intake, with a target average increase of 0·5 serves/d (37·5 g).

## Methods

### Study design

This study used the MOST framework to develop a package of initiatives to increase children’s vegetable intake in LDC centres. Details are outlined in a study protocol paper^(27)^ (·https://doi.org/10·1136/bmjopen-2020-047618). This article reports Phase 2 of the MOST framework, the optimisation phase. A 12-week, eight-condition randomised factorial experiment was conducted to test the main and synergistic effects of three initiatives targeting centre food provision, staff mealtime practices (mealtime environment) and a vegetable-focused sensory-based curriculum at improving children’s vegetable intake in LDC centres. The trial was registered *a priori* with the Australia and New Zealand Clinical Trials Registry (ACTRN12620001301954). The study follows the CONsolidated Standards of Reporting Trials guidelines for randomised trials.

### Setting and sample

LDC centres in metropolitan Adelaide, South Australia were recruited for the study between November 2020 and March 2021 by the study coordinator (DZ) and research assistants. Accredited LDC centres and LDC providers were contacted using a sampling list sourced from the Australian Children’s Education and Care Quality Authority website^(28)^. Centres were randomly sampled from the sampling list, stratified by centre size (≤ 50 and > 50 enrolments) and socio-economic status (low, deciles 1–4, middle, deciles 5–7, high, deciles 8–10) using the Index of Relative Socio-Economic Advantage and Disadvantage Socio-Economic Index for Areas^(29)^, and invited to participate in the study. Interested centres were screened for eligibility. Eligible centres were those that operated for at least 8 h Monday to Friday, prepared food onsite, served lunch and two between-meal snacks each day (i.e. known in Australia as morning and afternoon tea) and enrolled children aged 2–5 years. Children aged 2–5 years who were present on data collection days were eligible to participate. Children with severe allergies or medical conditions that prevented them from consuming the standard centre menu were excluded. Participating centre staff provided informed consent for their centre (directors) and themselves (directors, cooks, educators and teachers). Parents of children enrolled at the centre were provided with an information sheet and opt out consent form prior to baseline and follow-up data collection via the centre’s usual communication channels, allowing them to opt out of the study on behalf of their child.

### Randomisation, intervention conditions and initiatives

Following consent, centres were randomised to one of eight experimental conditions (seven intervention groups and one waitlist control group who received access to the intervention materials following completion of follow-up data collection, see Table 1). Cluster randomisation was used, in which children attending the same centre were assigned to the same intervention. The eight conditions reflected all possible combinations of initiatives (Table 1). Random allocation was conducted using sequence generation in GraphPad after the completion of baseline data collection using stratification according to centre size (small < 50 children or large > 50 children) and Socio-Economic Indexes for Areas (low, middle, high)^(29)^ by a researcher not involved in the study (BJ), to obtain equal representation from each group. Participating centres, families and research staff were blinded to group allocation at baseline only.

**Table 1:** The eight different combinations of initiatives (conditions) allocated to the study groups

Group	1. Food provision	2. Mealtime environment	3. Curriculum
1 (control)	–	–	–
2	–	–	✓
3	–	✓	✓
4	✓	–	✓
5	✓	–	–
6	✓	✓	–
7	–	✓	–
8	✓	✓	✓

✓ = allocated to the initiative, – = not allocated to the initiative.

### Intervention conditions and initiatives

Centres randomly allocated to the control group (*n* 1) were instructed to continue their usual practice. Educators and cooks were explicitly asked not to complete any nutrition training (excluding allergy or food safety training) and to not use any vegetable or nutrition curriculum at the centre during the 12 weeks. Control centres were offered access to the initiatives at the completion of follow-up data collection. The intervention groups (*n* 7) trialled a combination of the three initiatives ((1) *food provision*, (2) *mealtime environment*, (3) *curriculum*) for 12 weeks, comprising a 4-week preparation phase and an 8-week implementation phase. The preparation phase enabled staff to complete the online trainings, prepare for the curriculum implementation and complete the menu assessment (described below). The implementation phase involved delivery of the initiatives, described in detail in the protocol paper^(27)^ and in brief below.

#### Food provision initiative

The *food provision* initiative comprised an online training module and online menu assessment tool for centre cooks to support them to increase the provision of vegetables across all eating occasions. The online training took approximately 45–55 min to complete and covered topics such as menu planning and the importance of healthy eating. The menu assessment tool (FoodChecker; https://foodchecker.heas.health.vic.gov.au/) compared compliance of the centre menu with the Healthy Eating Advisory Service Menu Planning Guidelines for LDC^(30)^ and provided recommendations to meet menu guidelines for each food group^(30)^, for example, ‘add 600 g of vegetables or legumes to morning tea, lunch or afternoon tea’. For helpful ideas, see ‘*Making veggies fun for kids’*. Cooks were asked to complete the training and menu assessment and revise their menu according to the recommendations provided within the 4 weeks of the preparation phase, before implementing the revised menu in the 8-week implementation phase.

#### Mealtime environment initiative

The *mealtime environment* initiative comprised an online interactive mealtime training module for centre educators (promoting healthy eating in LDC https://heas.health.vic.gov.au/training/training-early-childhood-sector) to support them to use mealtime practices that promote children’s vegetable acceptance and intake. The interactive training took approximately 45–55 min to complete and aimed to increase educators’ knowledge and skills to use feeding practices at mealtimes that promote vegetable familiarisation and opportunities to try vegetables.

#### Curriculum initiative

The *curriculum* initiative comprised a curriculum package for centre educators that aims to provide opportunities for children to learn about, try and enjoy vegetables by increasing their exposure to a variety of familiar and unfamiliar vegetables. The curriculum included sixteen lessons (approximately 20 min each) and sixteen snack time activities (approximately 10 min each) (online Supplementary Table 1). The curriculum is based on experiential learning, sensory education and insights on vegetable preference development in children. It is based on the same principles as the evidence-based Taste & Learn^TM^ vegetable education curriculum for primary school children (aged 5–12 years)^(31,32)^, with activities suitable for younger children. Vegetables were tasted during each activity and specific vegetable suggestions (familiar and unfamiliar, raw and cooked) were provided for each lesson, which could be tracked using a ‘Vegetable Adventure Chart’.

As a support strategy for the three initiatives, weekly email and/or text message reminders were sent over the 12-week implementation period to assist with initiative compliance. Directors were sent a weekly email update about the relevant initiatives and participating staff were sent short message service text messages, for example, ‘this week the lessons are ‘The five senses: sound and texture’ (for the *Curriculum* initiative) and ‘*We hope that you have found the training informative and gained skills to support children to eat vegetables at mealtimes*’ (for the *Mealtime environment* initiative)’.

### Data collection and entry

Data were collected on two occasions: (i) at baseline, pre-initiative implementation, between February and April 2021 and (ii) at follow-up, post-intervention approximately 12 weeks later, between June and September 2021. The primary outcome was children’s vegetable intake, g/d. Secondary outcomes were vegetable provision and waste (used to calculate intake), and initiative fidelity and acceptability.

#### Centre, staff and child socio-demographic characteristics

Data on centre and staff characteristics were collected via questionnaires completed by the director and participating staff, respectively, and are presented in Table 2. Data on child characteristics were collected by research staff at data collection days by obtaining the relevant room/s attendance list and requesting additional information from educators.

**Table 2:** Centre (*n* 32), child (*n* 1039) and staff (*n* 276) characteristics†

	1. Control		2. Curriculum		3. Curriculum + mealtime environment		4. Curriculum + food provision		5. Food provision		6. Food provision + mealtime environment		7. Mealtime environment		8. Curriculum + food provision + mealtime environment		Total	
Characteristic	*n*	%	*n*	%	*n*	%	*n*	%	*n*	%	*n*	%	*n*	%	*n*	%	*n*	%
Centre characteristics																		
No. of centres	4		4		4		4		4		4		4		4		32	
Centre SEIFA decile*:																		
Low (1–4)	2	50	1	25	2	50	2	50	2	50	2	50	1	25	0	0	12	38
Middle (5–7)	2	50	1	25	1	25	1	25	0	0	1	25	1	25	2	50	9	28
High (8–10)	0	0	2	50	1	25	1	25	2	50	1	25	2	50	2	50	11	34
Centre type:																		
Private	4	100	4	100	3	75	3	75	4	100	3	75	3	75	4	100	28	87·5
Community	0	0	0	0	1	25	1	25	0	0	1	25	1	25	0	0	4	12·5
No. of children enrolled																		
Median	108		97		88		77		80		77		87		79		87	
IQR	33–130		90–100		46–176		53–106		59–84·0		50–120		70–120		34–150		33–176	
No. of staff																		
Median	30		16		25		30		26		22		18		19		24	
IQR	14–55		11–36		17–41		20–33		22–37		15–25		11–34		9–28		9–55	
No. of cooks																		
Median	1		1		1		1		1		2		2		1		1	
IQR	1–1		1–1		1–2		1–1		1–1		1–2		1–2		1–1		1–2	
No. of centres with kitchen assistants	0	0	1	25	1	25	0	0	0	0	0	0	0	0	0	0	2	6
No. of educators employed																		
Certificate level																		
Median	28		11		22		27		24		19		15		16		20	
IQR	12–52		5–31		16–37		18–30		20–35		13–23		9–29		7–25		5–52	
Bachelor level																		
Median	2		4		2		2		1		1		1		2		1	
IQR	1–2		1–6		0–2		1–2		1–2		1–2		1–3		1–2		0–6	
Centre has a nutrition policy (yes)	4	100	4	100	3	75	3	75	3	75	3	75	4	100	4	100	28	88
Centre has a policy for dietary requirements (yes)	4	100	4	100	4	100	2	50	4	100	3	75	4	100	4	100	29	90
Child characteristics																		
No. of children	129		105		143		162		144		122		121		113		1039	
Age (months):																		
Median	32·0		40·0		36·0		39·0		40·0		40·5		36·0		43·0		38	
IQR	26·0–42·0		33·0–45·0		30·0–43·0		30·0–48·0		30·0–48·0		34·8–48·0		31·0–44·0		36·0–50·0		30·0–46·0	
Gender: (male)	63	48·8	64	61·0	69	48·3	79	48·8	75	52·1	69	56·6	62	51·2	63	55·8	544	52·4
ATSI:	2	1·6	2	1·9	1	0·7	1	0·6	2	1·4	3	2·5	2	1·7	0	0·0	13	1·3
Dietary requirement (yes)	15	11·6	8	7·6	8	5·6	17	10·5	7	4·9	8	6·6	11	9·1	16	14·2	90	8·7
Staff characteristics																		
No. of staff (total)	34		32		37		40		30		35		38		30		276	
No. of cooks	4		5		5		4		4		7		5		5		39	
No. of educators	28		21		25		31		22		20		22		15		184	
No. of teachers	2		6		7		5		4		8		11		10		53	
Age (years)																		
Median	33·0		34·0		33·5		32·5		34·0		39·0		37·0		38·0		35·0	
IQR	25·8–44·2		24·0–44·8		26·8–42·2		26·0–40·8		22·2–40·5		29·0–47·0		29·0–43·0		26·0–48·0		26·0–43·0	
Female	32	94·1	28	87·5	36	97·3	36	90·0	26	86·7	33	94·3	34	89·5	28	93·3	253	91·7
Total time worked in childcare (months)																		
Median	65·8		35·9		47·9		47·9		71·8		119·7		59·8		47·9		59·8	
IQR	20·9–119·7		12·0–95·7		18·0–140·6		23·9–83·8		29·9–119·7		59·8–239·3		23·9–137·6		22·6–95·7		23·9–122·7	

ATSI, Aboriginal or Torres Strait Islander, IQR; inter-quartile range; SEIFA, Socio-Economic Index for Areas.

* As per ABS classification of postcode ranking 2016.

† Data presented as *n* (%) or median (IQR).

#### Primary outcome: children’s vegetable intake

Children’s dietary intake was measured by trained research staff at baseline and follow-up using the weighed plate wastage method, the gold standard for individual food consumption. This method has previously been used in studies in the LDC setting^(33–36)^. Dietary intake data were collected using a standardised procedure across 2 d (to capture children of different ages in different rooms, e.g. 2–3 years and 3–5 years) for all eligible children in attendance. Data were collected for one meal (i.e. lunch) and two between-meal snacks (i.e. morning tea and afternoon tea) including drinks at mealtimes, excluding water. All bowls and cups were labelled with ID stickers and each individual plated food component (e.g. bread, pasta with sauce, milk), including additional servings and leftovers, was weighed to the nearest 0·1 g using calibrated digital scales. Leftovers included any food dropped from the child’s plate onto the floor. Centre staff were informed about the study and mealtimes were encouraged to continue as normal. Research staff avoided interaction with staff and children during the data collection period to reduce any influence on food intake. Details of the foods provided, including recipes, types and brands of foods, were obtained from the centre cook.

All foods were entered into Excel and an eight-digit food code was assigned to individual food items (e.g. wholemeal bread) using the AUSNUT 2011–2013 database^(37,38)^. Coding decisions were made by research staff and discussed and approved by the chief investigator. Ten percentage of entries were double coded to ensure consistency between research assistants. For mixed meals (e.g. spaghetti bolognaise), recipes were entered into FoodWorks Professional Version 10 (Xyris Software) using a standard protocol developed for this study. The ratio of the ingredient weight (e.g. minced beef) to the total recipe weight was used to calculate the proportion of each ingredient served and consumed. The AUSNUT database was used to calculate weight (in g) of intake by food group (grains, vegetables, fruit, dairy, meat and alternatives, fats and oils, discretionary foods) for each item. Based on the eight-digit code assigned to each food item, food group provision and waste in g per child per day were calculated. The amount of food consumed was subsequently calculated by subtracting the mass of the food waste left over from the initial mass of food provided. As the primary outcome of this study was vegetable intake, only data relating to the vegetable food group are presented from this point forward. Vegetable intake, as defined by the Australian Guide to Healthy Eating, included dark green or cruciferous vegetables (e.g. broccoli), root/tubular/bulb vegetables (e.g. potato, carrot, onion), legumes/beans (e.g. chickpeas, tofu) and other vegetables (e.g. tomato, zucchini, mushrooms), as well as vegetables in mixed dishes (i.e. recipes)^(39)^. Vegetable intake (g) was calculated as provision (g) – waste (g) and converted to standard servings (1 serve = 75 g vegetables) according to The Australian Guide to Healthy Eating^(39)^.

#### Secondary outcomes: initiative fidelity and acceptability

The extent to which the initiatives were delivered as planned (fidelity) and acceptability of the initiatives by cooks and educators were assessed at follow-up using staff-completed paper or online questionnaires. Outcomes were the proportion of participating cooks and educators that completed the trainings, completed the menu assessment and delivered the curriculum, and the proportion of initiative components delivered to children. The average proportion of cooks, educators and teachers who completed the initiative components was determined for each intervention condition. Fidelity of the curriculum initiative was determined using an educator-completed checklist of lessons and snack time activities delivered. Reasons for non-completion of the initiatives were collected in the cook’s and educator’s follow-up questionnaires and collated and analysed to identify the most common themes. Acceptability of each of the initiatives was assessed using a purposefully designed set of evaluation questions based on the Learning Object Review Instrument^(40)^ included in the cook and educator follow-up questionnaires. The items covered the domains of: (i) content quality, (ii) learning goal alignment, (iii) motivation, (iv) interaction useability, (v) presentation design and (vi) accessibility and were rated using a 5-point Likert scale from strongly disagree to strongly agree.

### Statistical analysis

All statistical analyses were conducted using R version 4.1.1 (R Foundation for Statistical Computing) by a statistician not involved in study implementation. All data were checked and cleaned prior to analyses. Socio-demographic data are reported as frequency (%) or median (inter-quartile range). Vegetable provision, waste and intake data (g/d) are reported descriptively as median (inter-quartile range) for each group at follow-up. Fidelity of the three initiatives is reported as median (inter-quartile range) proportion of staff completing each initiative component according to each of the eight conditions. Acceptability of the three initiatives is reported as the number of respondents who agreed or strongly agreed with the Learning Object Review Instrument framework statements.

The optimised combination of initiatives for increasing children’s vegetable intake was determined using a linear mixed-effects model with the three intervention initiatives (food provision, mealtime environment, and curriculum) and their 2-way and 3-way interaction terms. A backward stepwise approach was performed based on Type III Sum of Squares, where the least significant interaction term was removed at each step using a *P*-value threshold of 0·05. Centre size and socio-economic status (Socio-Economic Index for Areas), and child age and gender, were included as covariates for adjustment. A random effect with rooms nested within sites, allowing the variance to differ across intervention groups, was used to account for cluster randomisation by site. In contrast to the pre-post analysis plan outlined in the protocol^(27)^, baseline vegetable intake (online Supplementary Table 2) was not included in the analysis due to 40 % of children at follow-up not being present at baseline because of children changing rooms, attendance on different days or children newly enrolled at the centre. Vegetable intake at follow-up was log-transformed to achieve model assumptions, and the geometric means for each group was reported along with corresponding 95 % CI. Post-hoc analyses were conducted comparing the geometric mean of vegetable intake for each group to the control group and the ratio of the geometric means (95 % CI) was reported. The group with the greatest ratio of geometric mean (95 % CI) was determined to be the most optimised combination of initiatives, while considering initiative fidelity and acceptability.

## Results

### Sample characteristics

**Fig. 1:**
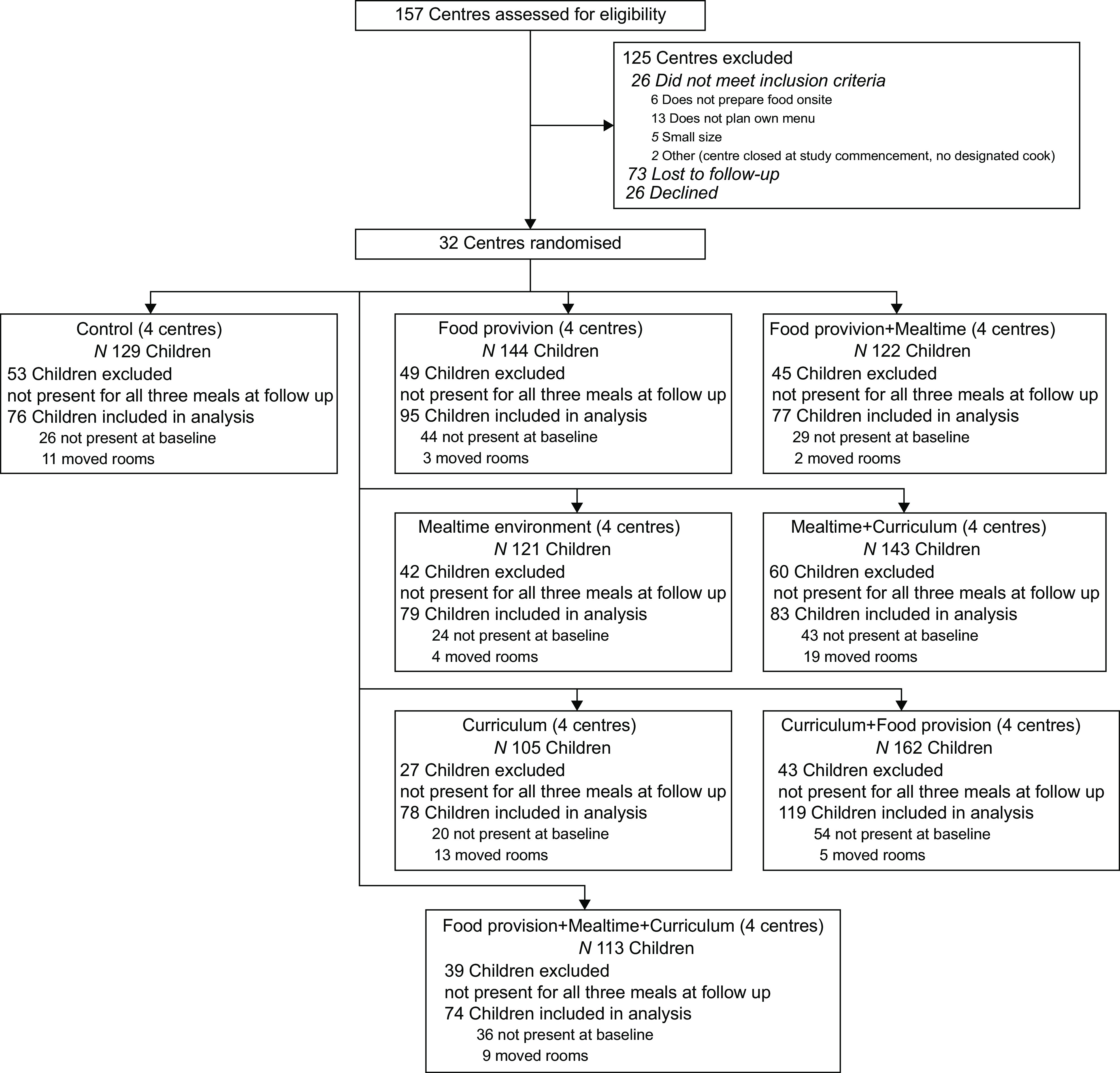
Flow diagram of centres and children through the study according to the CONsolidated Standards of Reporting Trials (CONSORT)

Figure 1 shows the flow of centres and children through the study. There were 157 centres assessed for eligibility and thirty-two centres randomised to one of the eight groups with four centres in each group. Data were collected for 1039 children across the eight groups of which 358 were excluded from the final analysis due to not being present for all three meals (total, *n* 681; *n* 74–119 per condition). Participating staff (*n* 276) included 184 educators and fifty-three teachers (henceforth, collectively referred to as educators) and thirty-nine cooks. Centre, child and staff characteristics are presented in Table 2.

### Primary outcome: children’s vegetable intake

Overall, there were no statistically significant two-way (*P* > 0·05) or three-way interactions (cook: educator: curriculum *P* = 0·29) between initiatives, indicating that the effect of one initiative did not depend on another. Therefore, in the final main effects model comparisons were performed between each of the three initiatives instead of the eight intervention conditions. The main effects analysis of each of the initiatives found no statistically significant differences in vegetable intake when compared with control (i.e. no initiative) for children receiving the *mealtime environment* initiative (ratio of geometric mean 1·77 (95 % CI 0·90, 3·48), *P* = 0·09), *curriculum* initiative (1·54 (95 % CI 0·78, 3·04), *P* = 0·21) and the *food provision* initiative (0·73 (95 % CI 0·37, 1·43), *P* = 0·34), (online Supplementary Table 3).

Post-hoc comparisons showed that there were also no statistically significant differences in vegetable intake for any of the seven intervention groups when compared with the control group (ratio of geometric mean ranged from 0·66 to 3·29) (*P* > 0·05) (Table 3). Of the seven intervention groups, the effect on vegetable intake was greatest for the combined *curriculum with mealtime environment* initiative where, on average, vegetable intake was 3·29 times higher than the control group (ratio of geometric mean 3·29 (95 % CI 0·96, 11·27), *P* = 0·06). The difference in vegetable intake between the two groups was on average 26·69 g/d (38·32 g–11·63 g) per child, equivalent to 0·36 more serves of vegetables consumed by children in the *curriculum with mealtime environment* initiative condition than in the control group. In comparison, vegetable intake by children receiving all three initiatives (*curriculum with food provision with mealtime environment*) was 1·34 times higher than that of control children (ratio of geometric mean 1·34 (95 % CI 0·36, 5·08), *P* = 0·65), equivalent to a 4 g (15·63–11·63 g) difference or 0·05 serve difference per child per day.

**Table 3:** Children’s vegetable (g/d) provision, waste and intake for the eight groups at follow-up (*n* 681)

Group	*n*	Provision		Waste		Intake		Geometric mean (intake)	95 % CI	Ratio of geometric mean (intake)	95 % CI	*P*
		Median	IQR	Median	IQR	Median	IQR					
1. Control	76	35·5	16·4–63·6	12·7	3·0–26·1	18·1	7·3–35·3	11·63	4·73, 28·57	Reference		
2. Curriculum	78	67·6	46·5–107·1	25·8	12·4–36·1	39·6	19·4–75·8	26·22	10·32, 66·54	2·25	0·62, 8·22	0·21
3. Curriculum + mealtime environment	83	82·0	41·2–152·0	17·2	6·8–38·1	49·3	19·3–118·5	38·32	16·41, 89·42	3·29	0·96, 11·27	0·06
4. Curriculum + food provision	119	34·7	24·8–54·1	12·1	5·2–20·6	21·6	11·3–37·5	16·05	6·55, 39·30	1·38	0·39, 4·90	0·60
5. Food provision	95	61·2	38·8–95·6	27·0	9·1–43·3	29·7	8·8–60·1	7·72	3·10, 19·21	0·66	0·18, 2·39	0·51
6. Food provision + mealtime environment	77	78·2	48·9–95·5	29·6	11·7–42·2	38·8	14·5–71·4	30·43	12·15, 76·17	2·61	0·72, 9·45	0·14
7. Mealtime environment	79	70·3	40·6–107·7	32·7	12·7–43·2	38·8	6·1–69·7	18·41	7·13, 47·48	1·58	0·43, 5·82	0·47
8. Curriculum + food provision + mealtime environment	74	56·2	32·8–82·7	19·2	8·5–31·5	32·0	6·6–59·4	15·64	5·87, 41·62	1·34	0·36, 5·08	0·65

IQR, inter-quartile range.

Post-hoc comparisons of each of the initiatives compared with the control group (without adjustment for multiple testing), showing the effect (ratio of geometric means, 95 % CI and *P*-value) of each intervention condition compared with the control group, where *P* < 0·05 means there was evidence of interaction.

Three-way interaction (mealtime environment with curriculum with food provision) *P* = 0·29; 2-way interaction: mealtime environment with curriculum interaction *P* = 0·25; food provision with curriculum interaction *P* = 0·25; food provision with mealtime environment interaction *P* = 0·68.

### Secondary outcomes: vegetable provision and waste

Vegetable provision was greatest for the *curriculum with mealtime environment* initiative combination, with 82 g of vegetables provided per child per day (equivalent to 1·1 serves/child/day). In comparison, vegetable provision for the control group was on average 35·5 g per child per day (equivalent to 0·5 serves/child/day). Vegetable waste was 17·2 g per child per day and 12·7 g per child per day for the combined *curriculum with mealtime environment* initiatives condition and control group, respectively. In comparison, vegetable provision in the group receiving all three initiatives (*curriculum with food provision with mealtime environment*) was 56·2 g per child per day and waste was 19·2 g.

### Secondary outcome: initiative fidelity

Fidelity of the three initiatives for each of the eight conditions is shown in Table 4. Out of the three initiatives, the curriculum initiative had the highest completion rates, ranging from 92 to 98 % and 88 to 100 % for the lessons and snack time activities, respectively. For the *mealtime environment* initiative, the median proportion of educators completing the training ranged from 0 to 61 %. For the *food provision* initiative, the median proportion of cooks completing the training and menu assessment and revision ranged from 0 to 100 % and 0 to 50 %, respectively. Of the seven intervention initiative conditions, the combined *curriculum with mealtime environment* initiative condition had the highest fidelity for the mealtime environment educator training (61 %), while the majority of the curriculum (94 % and 100 % for the lessons and snack time activities, respectively) was also delivered.

**Table 4:** Fidelity of the three initiatives: median (IQR) proportion of staff completing each initiative component at the centre according to each condition

Group	Curriculum				Mealtime environment		Food provision			
	Lessons		Snack time activities		Educator training		Cook training		Menu assessment and revision	
	Median	IQR	Median	IQR	Median	IQR	Median	IQR	Median	IQR
1. Control	–		–		–		–		–	
2. Curriculum	93·8	31·2–100·0	98·4	18·8–100·0	–		–		–	
3. Curriculum + mealtime environment	93·8	34·4–100·0	100·0	50·0–100·0	60·7	0·0–87·5	–		–	
4. Curriculum + food provision	98·4	78·1–100·0	100·0	78·1–100·0	–		100·0	100·0–100·0	0·0	0·0–100·0
5. Food provision	–		–		–		100·0	0·0–100·0	50·0	0·0–100·0
6. Food provision + mealtime environment	–		–		0·0	0·0–100·0	0·0	0·0–100·0	25·0	0·0–100·0
7. Mealtime environment	–		–		25·0	0·0–100·0	–		–	
8. Curriculum + food provision + mealtime environment	92·2	12·5–100·0	100·0	0·0–100·0	28·6	0·0–75·0	75·0	0·0–100·0	0·0	0·0–100·0

The most common reason for educators not completing the mealtime environment training was that they were not aware of the training/the training was not facilitated (*n* 18) (data not shown). The most common reasons for non-completion of lesson delivery for the curriculum initiative were that other staff taught it (*n* 11), they were not in the room at the time/work part time (*n* 11) and that other lessons were planned (*n* 11). The most common reason for non-completion of the food provision initiative was a lack of time for both the cooks training and menu assessment and revision.

### Secondary outcome: initiative acceptability

Acceptability of the three initiatives according to participating educators, teachers and cooks is displayed in online Supplementary Table 4. Most educators/teachers (*n* 13/16) would recommend the curriculum to others and most (*n* 11/16) found the amount of preparation required to deliver the curriculum was reasonable. Nearly all educators found the mealtime training useful (*n* 34/38) and the duration of the training appropriate (*n* 34/38), with most (*n* 31/38) agreeing/strongly agreeing they would recommend it to others. Although most cooks (*n* 10/11) found the training useful, only two-thirds (*n* 7/11) would recommend it to others. The majority of cooks (*n* 8/11) found the duration of the training appropriate; however, few (*n* 3/10) found the duration of time to use the menu assessment tool reasonable. Only half of cooks (*n* 5/10) found the menu assessment tool useful and less than two-thirds (*n* 6/10) would recommend it to others.

## Discussion

This study used the MOST framework to systematically develop a package of initiatives to increase children’s vegetable intake in the LDC setting. The independent and synergistic effects of initiatives targeting centre food provision, staff mealtime practices and a child-focused curriculum were examined. Although there were no statistically significant differences in vegetable intake between any of the intervention groups and the control group, findings showed that the *curriculum with mealtime environment* initiative combination had the most promise for increasing children’s vegetable intake. That is, vegetable intake was on average three times greater (equivalent to 26·7 g or 0·36 more serves of vegetables per child per day) for children exposed to both the *curriculum and mealtime environment initiatives* than children in the control group not exposed to any initiative. On average, children in the *curriculum with mealtime environment* condition were provided with more than double the amount of vegetables than children in the control group (82 g/d *v* 36 g/d) with little increase in waste (17 g/d *v* 13 g/d). Initiative fidelity was greatest for the *curriculum* initiative (> 90 % completion rates) and good acceptability was reported for both the *curriculum and mealtime environment initiative*s, with four in five educators recommending these initiatives to others. Findings from this study suggest that interventions simultaneously targeting a combination of the curriculum and mealtime environment are the most efficient and effective targets for increasing children’s vegetable intake as opposed to a more complex intervention with all three initiatives. This combination will be taken forward to the next stage (evaluation phase) of the MOST framework to be evaluated in a randomised controlled trial design^(27)^.

Although the threshold for statistical significance was not reached, *P* = 0·06, for the difference in vegetable intake between the *curriculum and mealtime environment* group and control group, the effect size of 0·36 more serves of vegetables consumed per day for children exposed to this initiative combination is comparable to other interventions in LDC settings in Australia and was considered meaningful. For example, a recent childcare intervention to improve centre compliance with nutrition guidelines in New South Wales found a 0·4 serve (*P* < 0·001) increase in children’s (2–5 years) vegetable intake per day when compared with children in control centres^(36)^. In contrast, a multi-intervention South Australian study investigating the impact of the Start Right Eat Right intervention (educator and cooks training) on improving centre menus, policies and the eating environment reported a 0·1 serve (*P* = 0·083) increase in children’s (aged 2–4 years) vegetable intake per day compared with before the intervention but negated by an increase in waste^(34)^. The findings of this study are also comparable to those reported in systematic reviews of interventions across home, community and educational settings, which found an average short-term (3 month) increase in children’s (2–12 years) vegetable intake of approximately 30 %, equivalent to a 0·25–0·50 serve increase^(41,42)^. Importantly, any improvement in vegetable intake, regardless of size, is important for establishing a preference for vegetables and thus a 0·36 serve increase in vegetable intake, although not statistically significant, is promising and could make a meaningful contribution to improvement in public health nutrition^(43)^.

The factorial design used in this study allowed for identification that the synergistic effect of the *curriculum with mealtime environment* initiative combination was greater than the effect of these two initiatives independently. That is, vegetable intake was 3·29 times greater than the control group for the combination of the two initiatives compared with only 1·58 times (*mealtime environment)* and 2·25 times (*curriculum)* greater for the groups receiving these initiatives alone. In addition to this, exposure to only one of the initiatives resulted in lower vegetable provision (70 g and 68 g, *curriculum* and *mealtime environment,* respectively) and more waste (33 g and 25 g, respectively) than exposure to the combination of the two initiatives (82 g provided, 17 g waste). Together, these findings suggest that although the mealtime training provides educators with the *knowledge* to teach children to learn to enjoy vegetables, the curriculum resources provide them with the *tools* to do so.

Despite the hypothesis that the combination of all three initiatives would have the largest effect on children’s vegetable intake, the addition of the food provision initiative to the curriculum and mealtime Environment initiative combination did not have any added benefit. In fact, the effect on children’s vegetable intake was less for the *curriculum with mealtime environment with food provision* initiative combination than the c*urriculum with mealtime environment* initiative combination (1·34 and 3·29 times higher than the control group, respectively). This may suggest that more complex interventions could increase burden, acting as a barrier to implementation and effectiveness. Further, vegetable intake for the *food provision* initiative alone was lower than the control group (ratio of geometric mean 0·66 (95 % CI 0·18, 2·39)). The latter finding is supported by a review of healthy eating interventions in preschools and kindergartens which found that single component interventions were insufficient to significantly increase children’s vegetable intake^(44)^. The vegetable provision findings also demonstrate why the hypothesis was not supported, with all groups receiving the *food provision* initiative (with or without other initiatives) providing less vegetables (56–78 g) than the combined *curriculum with mealtime environment* group (82 g). Overall, these findings suggest that the synergistic effect of the *curriculum with mealtime environment* initiative combination led to greater provision and intake of vegetables, and less waste, than each of the three initiatives independently.

The fidelity and acceptability outcomes support the vegetable intake findings for the three initiatives. That is, fidelity and acceptability were higher for the curriculum and mealtime initiatives than the food provision initiative. For the curriculum initiative, the majority of lessons (92–98 %) and snack-time activities (98–100 %) were delivered as planned and when combined with the mealtime environment initiative, the number of educators completing the mealtime training was highest (61 %). In contrast, fidelity was low for the menu assessment and revision component (0–50 % completion across groups) of the food provision initiative, which, if implemented correctly, is expected to lead to changes in menu provision of vegetables to meet recommended guidelines. These low completion rates are unsurprising given the poor acceptability of the menu assessment tool (FoodChecker), whereby only half of cooks would recommend it to others. This is likely due to most cooks not agreeing that the duration of time to use the menu assessment tool was reasonable. Further, only half of cooks agreed that the menu assessment tool supported them to provide vegetables on the menu in line with the menu planning guidelines. This may be due to the feedback provided not being specific enough, for example, not providing guidance on what ‘600 grams of vegetables or legumes’ is in practical terms (i.e. in cups).

The acceptability findings also showed that four out of five educators would recommend the curriculum and mealtime initiatives to others, whereas only two-thirds of cooks would recommend the cooks training to others. The food provision initiative was completed solely by the centre cook, in contrast to delivery of the curriculum being a shared task between educators and may explain the higher fidelity results for the curriculum initiative compared with the food provision initiative. Further to this, educators/teachers get assigned programming time within which to plan the curriculum and/or to undertake trainings, whereas insufficient time has been a commonly reported barrier by cooks to implementing dietary menu guidelines^(45)^. Thus, the fidelity and acceptability of initiatives in LDC centres play a key role in the effectiveness of the initiatives to improve vegetable intake and must be key considerations for adoption into practice. The findings from this study suggest that educator-targeted and child-targeted interventions may be more feasible for adoption within current LDC setting practices than cook-targeted interventions. Future interventions targeting centre cooks may be more effective when paired with implementation support strategies targeting barriers to adoption, such as management support. Consideration could be made to future reforms of the time allocated to cooks to undertake their responsibilities, including prioritisation of menu planning, and simpler training to ensure cooks provide adequate vegetables to support the educator- and child-targeted interventions.

A major strength of this study was the use of the MOST framework and optimising the initiative package using a factorial experimental design. The MOST framework is ideal for not only evaluating but also optimising behavioural interventions^(26)^, an approach that supports adoption and feasibility of interventions into practice^(23)^. The factorial experimental design of the optimisation phase provided an efficient way to assess the initiatives independently and in combination to determine the feasibility of components prior to testing them with a fully powered experiment^(46)^. It also facilitated the understanding of the most optimised initiative package to increase children’s vegetable intake in LDC centres. This approach was less resource intensive than, for example, an 8-group randomised controlled trial and supports adoption into practice by only taking forward intervention components that demonstrate effectiveness. Further to this, the fidelity and acceptability findings from the process evaluation have highlighted barriers to implementation and thus effectiveness. However, the study is not without limitations. Children’s vegetable intake data were collected on 1 d only and, therefore, may not be representative of usual intake as children’s intake can vary due to a number of factors including what is served on the day and individual preferences. However, data collection was undertaken on multiple days per centre to capture variation in vegetable content on the menu. There was also an adjustment to the pre-post analysis plan outlined in the published protocol^(27)^, whereby baseline vegetable intake was not included in the analyses because of a high turnover of children between baseline and follow-up and therefore a high proportion of missing paired data (40 %). Thus, given the analysis was conducted on follow-up data only without adjustment for baseline data, the findings should be interpreted with caution. Lastly, with some centres managed by the same childcare provider, the risk of contamination of initiatives across these centres cannot be discounted.

In conclusion, this study used the MOST framework to identify the most effective intervention components for improving children’s vegetable intake in LDC settings. Interventions simultaneously targeting both the curriculum and mealtime environment demonstrated the most promise for increasing children’s vegetable intake compared with a more complex intervention. Combined with the fidelity and acceptability findings, this study supports taking forward the *curriculum with mealtime environment* initiative combination to be evaluated in a randomised controlled trial as per the final stage of the MOST framework, the evaluation phase.

## Supplementary material

For supplementary material accompanying this paper visit https://doi.org/10.1017/S136898002300174X
